# Computational Fractional Flow Reserve From Coronary Computed Tomography Angiography—Optical Coherence Tomography Fusion Images in Assessing Functionally Significant Coronary Stenosis

**DOI:** 10.3389/fcvm.2022.925414

**Published:** 2022-06-13

**Authors:** Yong-Joon Lee, Young Woo Kim, Jinyong Ha, Minug Kim, Giulio Guagliumi, Juan F. Granada, Seul-Gee Lee, Jung-Jae Lee, Yun-Kyeong Cho, Hyuck Jun Yoon, Jung Hee Lee, Ung Kim, Ji-Yong Jang, Seung-Jin Oh, Seung-Jun Lee, Sung-Jin Hong, Chul-Min Ahn, Byeong-Keuk Kim, Hyuk-Jae Chang, Young-Guk Ko, Donghoon Choi, Myeong-Ki Hong, Yangsoo Jang, Joon Sang Lee, Jung-Sun Kim

**Affiliations:** ^1^Division of Cardiology, Severance Cardiovascular Hospital, Yonsei University College of Medicine, Seoul, South Korea; ^2^Department of Mechanical Engineering, Yonsei University, Seoul, South Korea; ^3^Department of Electrical Engineering, Sejong University, Seoul, South Korea; ^4^Department of Cardiovascular, Ospedale Papa Giovanni XXIII, Bergamo, Italy; ^5^Cardiovascular Research Foundation, Columbia University Medical Center, New York, NY, United States; ^6^Yonsei Cardiovascular Research Institute, Yonsei University College of Medicine, Seoul, South Korea; ^7^Department of Cardiology, Keimyung University Dongsan Hospital, Daegu, South Korea; ^8^Division of Cardiology, Yeungnam University Medical Center, Yeungnam University College of Medicine, Daegu, South Korea; ^9^National Health Insurance Service Ilsan Hospital, Goyang, South Korea; ^10^Division of Cardiology, CHA Bundang Medical Center, CHA University College of Medicine, Seongnam, South Korea

**Keywords:** fractional flow reserve (FFR), coronary computed tomography angiography (coronary CTA), optical coherence tomography (OCT), fusion image, computational fluid dynamics (CFD)

## Abstract

**Background:**

Coronary computed tomography angiography (CTA) and optical coherence tomography (OCT) provide additional functional information beyond the anatomy by applying computational fluid dynamics (CFD). This study sought to evaluate a novel approach for estimating computational fractional flow reserve (FFR) from coronary CTA-OCT fusion images.

**Methods:**

Among patients who underwent coronary CTA, 148 patients who underwent both pressure wire-based FFR measurement and OCT during angiography to evaluate intermediate stenosis in the left anterior descending artery were included from the prospective registry. Coronary CTA-OCT fusion images were created, and CFD was applied to estimate computational FFR. Based on pressure wire-based FFR as a reference, the diagnostic performance of Fusion-FFR was compared with that of CT-FFR and OCT-FFR.

**Results:**

Fusion-FFR was strongly correlated with FFR (*r* = 0.836, *P* < 0.001). Correlation between FFR and Fusion-FFR was stronger than that between FFR and CT-FFR (*r* = 0.682, *P* < 0.001; z statistic, 5.42, *P* < 0.001) and between FFR and OCT-FFR (*r* = 0.705, *P* < 0.001; z statistic, 4.38, *P* < 0.001). Area under the receiver operating characteristics curve to assess functionally significant stenosis was higher for Fusion-FFR than for CT-FFR (0.90 vs. 0.83, *P* = 0.024) and OCT-FFR (0.90 vs. 0.83, *P* = 0.043). Fusion-FFR exhibited 84.5% accuracy, 84.6% sensitivity, 84.3% specificity, 80.9% positive predictive value, and 87.5% negative predictive value. Especially accuracy, specificity, and positive predictive value were superior for Fusion-FFR than for CT-FFR (73.0%, *P* = 0.007; 61.4%, *P* < 0.001; 64.0%, *P* < 0.001) and OCT-FFR (75.7%, *P* = 0.021; 73.5%, *P* = 0.020; 69.9%, *P* = 0.012).

**Conclusion:**

CFD-based computational FFR from coronary CTA-OCT fusion images provided more accurate functional information than coronary CTA or OCT alone.

**Clinical Trial Registration:**

[www.ClinicalTrials.gov], identifier [NCT03298282].

## Introduction

Deciding whether to recommend coronary revascularization to patients with chest pain and intermediate stenosis on coronary angiography is challenging (1, 2). Therefore, in addition to the anatomical assessment of coronary stenosis, functional assessment is essential to evaluate the presence of myocardial ischemia, particularly in the setting of intermediate stenosis (1–5). Pressure wire-based fractional flow reserve (FFR) has been considered the gold standard for functional assessment of intermediate stenosis; it helps reduce unnecessary revascularization procedures (6, 7). Coronary computed tomography angiography (CTA) is a widely used non-invasive method for visualizing the coronary artery, and intravascular optical coherence tomography (OCT) has been used for accurate anatomical assessment of coronary stenotic lesions during angiography with exceptional higher resolution than intravascular ultrasound (8–10). In addition, computational fluid dynamics (CFD) has been applied to estimate computational FFR from coronary CTA- or OCT-based three-dimensional coronary model without using additional pressure guide wires or hyperemic agents (11–14). However, no studies have heretofore evaluated the usefulness of fusion images generated from both coronary CTA and OCT in clinical practice. Since many patients undergo coronary CTA to evaluate suspected coronary artery disease before being referred for angiography, it is hypothesized that if not only OCT but also coronary CTA images are available, the coronary CTA-OCT fusion images can be created and can provide more reliable information about coronary stenosis by combining delicate vessel curvature found in coronary CTA images with accurate lumen contour found in OCT images (8, 9, 15). Thus, this study aimed to present a novel approach for estimating CFD-based computational FFR from coronary CTA-OCT fusion images (Fusion-FFR). Pressure wire-based FFR was used as a reference to assess the diagnostic performance of Fusion-FFR as well as FFR derived from coronary CTA or OCT alone (CT-FFR or OCT-FFR) in patients with intermediate coronary stenosis.

## Materials and Methods

### Subjects and Study Design

The Integrated Coronary Multicenter Imaging Registry is a collaboration between four institutions in South Korea, created to evaluate the clinical impact of anatomical information from coronary CTA and OCT, as well as the functional information from FFR in patients with intermediate coronary stenosis with clinical follow-up (ClinicalTrials.gov, Identifier: NCT03298282). This study complied with the principles of the Declaration of Helsinki, and the Institutional Review Board at each participating center approved this study protocol. Written informed consent was obtained from all patients. Briefly, among patients who underwent coronary CTA for chest pain before being referred for coronary angiography, a total of 180 patients who had undergone both pressure wire-based FFR measurement and OCT examination during angiography to evaluate intermediate stenosis (40–70%) in any coronary artery were enrolled between November 2017 and June 2019 (the detailed inclusion and exclusion criteria are presented in Supplementary Table 1). Of these, 32 patients were excluded due to no intermediate stenosis of the left anterior descending artery (LAD) (*n* = 18), poor image quality of coronary CTA or OCT (*n* = 9), and incomplete OCT coverage (*n* = 5). Consequently, a total of 148 patients with intermediate stenosis on LAD were included in this study, and pressure wire-based FFR was used as a reference to assess the diagnostic performance of CFD- based computational FFRs (Supplementary Figure 1). The primary outcome was the correlation between pressure wire-based FFR and computational FFRs. The secondary outcome was the diagnostic performance of computational FFRs in assessing functionally significant stenosis. The correlation and diagnostic performance of Fusion-FFR were compared to those of CT-FFR and OCT-FFR.

### Image Acquisition, Analysis, and Fractional Flow Reserve Measurement

All subjects underwent coronary CTA before coronary angiography. Coronary CTA performance and acquisition of CTA images (64− or higher detector row scanners with prospective or retrospective electrocardiographic gating) were in accordance with the Society of Cardiovascular Computed Tomography guidelines (16). OCT imaging of the target lesion was performed using a frequency-domain OCT system (C7-XR OCT imaging system, LightLab Imaging Inc., St. Jude Medical, MN, United States). Cross-sectional OCT images were generated at a rotational speed of 100 frames/s. The fiber probe was withdrawn at 20 mm/s within the stationary imaging sheath.

All quantitative coronary angiography (QCA) and OCT analyses were performed at an independent core laboratory of Cardiovascular Research Center (Seoul, South Korea), and coronary CTA analysis was performed at Yonsei University CONNECT-AI Research Center (Seoul, South Korea) by experienced analysts who were blinded to the patient and procedure data. QCA analysis was performed using an off-line quantitative coronary angiographic system (CAAS, Pie Medical Instruments, Maastricht, Netherlands). Using the guiding catheter for magnification calibration, minimal lumen diameter was measured from diastolic frames in a single, matched view, showing the smallest lumen diameter. Coronary CTA analysis was performed using semi-automated image analysis software (QAngio CT RE, Medis Medical Imaging Systems, Leiden, Netherlands). Coronary CTA stenosis was evaluated by determining lumen diameter stenosis in each coronary segment ≥ 2 mm in diameter, using an 18-segment coronary model (12, 17). OCT analysis was performed using certified software (QIvus, Medis Medical Imaging Systems, Leiden, Netherlands). The reference lumen area was the region within the same segment as the lesion with the largest lumen. These reference area was proximal or distal to the stenotic area (usually within 10 mm of the stenosis, without major intervening branches). The minimal lumen area was identified at the segment with the smallest lumen area. Area stenosis was calculated as follows: [(mean reference lumen area - minimal lumen area) ÷ mean reference lumen area] × 100%. OCT-based plaque characteristics were also assessed; they are defined in Supplementary Table 2.

FFR was measured using a 0.014-inch pressure guidewire (St. Jude Medical, MN, United States). After equalizing process, the pressure guidewire was positioned distal to the target lesion. Hyperemia was induced by intravenous adenosine administered at 140 μg/kg/min *via* an antecubital vein. FFR was calculated as follows: mean hyperemic distal coronary pressure/mean aortic pressure. When FFR was ≤ 0.80, the stenotic lesion was considered functionally significant.

### Three-Dimensional Coronary Model Reconstruction and Image Fusion With Coronary Computed Tomography Angiography and Optical Coherence Tomography

Coronary CTA lumen contours at 0.25 mm intervals were manually extracted by experienced experts at the core laboratory. When coronary CTA lumen contours with side branches were extracted, the information regarding the direction of the side branches was also included. To create an OCT-derived three-dimensional coronary model for blood flow simulation, OCT lumen contours at 0.2 mm intervals were extracted using fully automated software (MATLAB, MathWorks, MA, United States), using the spatial continuity of the arterial walls in the transverse cross-sectional plane (18). A simple three-dimensional model was generated by eliminating side branches at bifurcations of the target lesions; thus, overall lumen contours were extracted by estimating the mother vessel lumen. The extracted lumen contour data were then used to create a three-dimensional model using semi-automated software designed in-house (Unity, Unity Technologies, CA, United States).

The entire image fusion process was performed as follows. Bifurcation directions of the coronary CTA and OCT lumens were identified as references. Coronary CTA lumens were then exchanged with the corresponding OCT lumens, while circumferential angulation and correction of the longitudinal location of the OCT lumens were applied to match the bifurcation direction of the target coronary CTA lumens. Since the lumen intervals differed for coronary CTA and OCT, OCT lumens were interpolated to obtain the same interval as the CTA lumens. Finally, the sizes of the remaining coronary CTA lumens were manually adjusted using the corresponding fusion model as a reference, and the fusion lumen data points were connected by ray casting to generate a meticulous three-dimensional coronary model.

### Computational Fluid Dynamics-Based Computational Fractional Flow Reserve Estimation

CFD-based blood flow simulation of reconstructed three-dimensional models was performed using the lattice Boltzmann method, which has been widely used for biofluidics; it uses lattice grids instead of complicated meshes for complex three-dimensional models (19, 20). Blood was modeled as an incompressible non-Newtonian fluid with a density of 1,060 kg/m^3^ based on the Carreau–Yasuda model (21). The inlet flow was designated as a steady flow condition based on the patient’s mean blood pressure assessed during coronary angiography. For the outlet boundary condition, a resistance model was used to reflect the circulatory resistance (22). Moreover, a no-slip boundary condition was used to calculate the interaction between the vessel wall and blood flow. Detailed equations and numerical methods used for estimating CFD-based computational FFR have been described previously (20). For each patient, Fusion-FFR, CT-FFR, and OCT-FFR were estimated. In addition, vorticity, helicity, and wall shear stress were also estimated and compared to support our hypothesis regarding the impact of vessel curvature of coronary CTA toward fusion images (23, 24).

### Statistical Analyses

Continuous variables were reported as means ± standard deviations or medians (interquartile ranges), and categorical variables were reported as numbers (percentages). Continuous variables were compared using Student’s *t*-test or Mann-Whitney test, as appropriate. Pearson correlation coefficient was calculated to evaluate the relationships between pressure wire-based FFR and computational FFRs (Fusion-FFR, CT-FFR, and OCT-FFR). The Bland–Altman analysis was also performed. Receiver operating characteristics (ROC) curve analysis was performed to evaluate the diagnostic performance of the computational FFRs in assessing functionally significant stenosis. The comparison between correlation coefficients was performed using Steiger’s *Z*-test, and the comparison between ROC curves was performed using DeLong’s test. The diagnostic accuracy, sensitivity, specificity, positive predictive value, and negative predictive values were also calculated as simple proportions with 95% confidence intervals (CIs), and the comparisons of these parameters were performed using R packages (R foundations for Statistical Computing, Vienna, Austria), including DTComPair. Other statistical analyses were performed using IBM SPSS, version 25.0 (IBM Corp., Armonk, NY, United States), and MedCalc, version 18.2.1 (MedCalc Software, Ostend, Belgium). All tests were two-sided, and a *P*-value < 0.05 was considered statistically significant.

## Results

### Baseline Characteristics and Computational Fractional Flow Reserve Estimation

**FIGURE 1:**
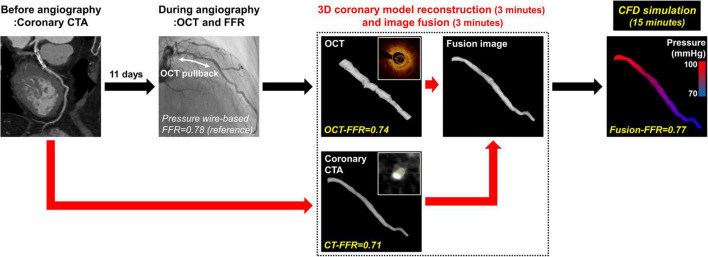
Overview of estimating CFD-based computational FFR from coronary CTA-OCT fusion images in patients with intermediate coronary stenosis. The current study evaluated a novel approach to estimate CFD-based computational FFR from coronary CTA-OCT fusion images in patients with intermediate coronary stenosis in the left anterior descending artery. CFD, computational fluid dynamics; CTA, computed tomography angiography; CT-FFR, computational FFR from coronary CTA; FFR, fractional flow reserve; Fusion-FFR, computational FFR from coronary CTA-OCT fusion images; OCT, optical coherence tomography; OCT-FFR, computational FFR from OCT.

The baseline clinical characteristics, coronary angiographic, coronary CTA, and OCT findings are presented in Tables 1, 2. The median time between coronary CTA and coronary angiography with OCT evaluation was 11 days (interquartile range: 4–18 days). Among 148 patients, 109 patients (73.6%) were male, and 44 patients (29.7%) presented with acute coronary syndrome. The LAD was considered as culprit vessel causing acute coronary syndrome in 21 patients (14.2%). There were bifurcation lesions with the side branch of ≥ 2.5 mm in diameter in 39 patients (26.4%). There were no complications during any of the procedures. The median pressure wire-based FFR at maximal hyperemia was 0.82 (interquartile range, 0.74–0.87). Functionally significant stenosis was observed in 65 patients (43.9%). The median CT-FFR was 0.78 (interquartile range, 0.72–0.84), OCT-FFR was 0.80 (interquartile range, 0.75–0.86), and Fusion-FFR was 0.81 (interquartile range, 0.74–0.85). A representative example of the three-dimensional coronary model reconstruction as well as the coronary CTA-OCT image fusion is presented in Figure 1. The three-dimensional model reconstruction for coronary CTA and OCT was completed within approximately 3 min. The entire image fusion with corresponding three-dimensional model reconstruction processes was completed within approximately 3 min, and estimation of Fusion-FFR using CFD was completed within approximately 15 min for each patient.

**TABLE 1:** Baseline clinical characteristics.

Variables	Total (*n* = 148)
Age (years)	63.4 ± 8.8
Male	109 (73.6)
Body mass index (kg/m^2^)	25.0 ± 2.9
Acute coronary syndrome	44 (29.7)
Hypertension	83 (56.1)
Diabetes mellitus	46 (31.1)
Dyslipidemia	72 (48.6)
Current smoker	33 (22.3)
Previous percutaneous coronary intervention	7 (4.7)

*Data are presented as the mean ± standard deviation (SD) or number (%).*

**TABLE 2:** Lesion characteristics.

Variables	Total (*n* = 148)
**Coronary angiography analysis**	
Reference vessel diameter (mm)	3.0 ± 0.5
Minimal lumen diameter (mm)	1.4 ± 0.5
Diameter stenosis (%)	53.9 ± 16.9
Lesion length (mm)	21.8 ± 9.8
Bifurcation lesions	39 (26.4)
**Pressure wire-based FFR measurement**	
FFR	0.82 (0.74–0.87)
FFR ≤ 0.8	65 (43.9)
**Coronary computed tomography angiography analysis**	
CTA stenosis (%)	61.1 ± 19.3
CTA stenosis ≥ 50%	107 (72.3)
Agatston score	283.7 ± 434.6
Agatston score ≥ 300	42 (28.4)
**Optical coherence tomography analysis**	
Proximal reference segment lumen area (mm^2^)	7.2 ± 2.6
Distal reference segment lumen area (mm^2^)	6.5 ± 3.1
Minimal lumen area of target lesion (mm^2^)	2.3 ± 1.2
Area stenosis (%)	84.3 ± 6.8
**Plaque morphology**	
Fibrous	28 (18.9)
Fibrocalcific	88 (59.5)
Lipid	64 (43.2)
Intimal vasculature	62 (41.9)
Cholesterol crystal	66 (44.6)
Calcific nodule	16 (10.8)
**CFD-based computational FFR estimation**	
Fusion-FFR	0.81 (0.74–0.85)
Fusion-FFR ≤ 0.8	68 (45.9)
CT-FFR	0.78 (0.72–0.84)
CT-FFR ≤ 0.8	89 (60.1)
OCT-FFR	0.80 (0.75–0.86)
OCT-FFR ≤ 0.8	73 (49.3)

*Data are presented as the mean ± SD, number (%), or median (interquartile range). CFD, computational fluid dynamics; CTA, computed tomography angiography; CT-FFR, computational FFR from coronary CTA; FFR, fractional flow reserve; Fusion-FFR, computational FFR from coronary CTA-OCT fusion images; OCT, optical coherence tomography; OCT-FFR, computational FFR from OCT.*

### Correlation and Diagnostic Performance of Computational Fractional Flow Reserves

**FIGURE 2:**
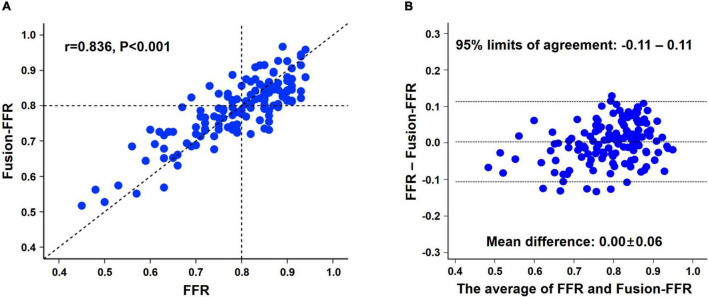
Relationship between pressure wire-based FFR and Fusion-FFR. Correlation **(A)** and agreement **(B)** between pressure wire-based FFR and Fusion-FFR. FFR, fractional flow reserve; Fusion-FFR, computational FFR from coronary CTA-OCT fusion images.

**FIGURE 3:**
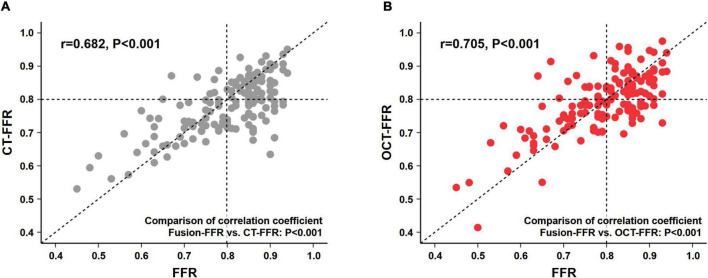
Correlation between pressure wire-based FFR and CFD-based computational FFR from coronary CTA or OCT images. Correlation between pressure wire-based FFR and CT-FFR **(A)**, and between FFR and OCT-FFR **(B)**. CFD, computational fluid dynamics; CTA, computed tomography angiography; CT-FFR, computational FFR from coronary CTA; FFR, fractional flow reserve; OCT, optical coherence tomography; OCT-FFR, computational FFR from OCT.

Fusion-FFR was strongly correlated with FFR (correlation coefficient, *r* = 0.836, *P* < 0.001; mean difference, 0.00 ± 0.06) (Figures 2A,B). Although CT-FFR was well correlated with FFR (*r* = 0.682, *P* < 0.001; mean difference, 0.02 ± 0.08), the correlation between FFR and Fusion-FFR was stronger (z statistic, 5.42, *P* < 0.001) (Figure 3A and Supplementary Figure 2A). Similarly, although OCT-FFR was well correlated with FFR (*r* = 0.705, *P* < 0.001; mean difference, 0.00 ± 0.07), the correlation between FFR and Fusion-FFR was stronger (z statistic, 4.38, *P* < 0.001) (Figure 3B and Supplementary Figure 2B). The correlation between FFR and CT-FFR was not different from that between FFR and OCT-FFR (z statistic, 0.58, *P* = 0.562). Anatomic variables were weakly correlated with FFR (percentage area stenosis on OCT: *r* = −0.451; percentage coronary CTA stenosis: *r* = −0.300).

**FIGURE 4:**
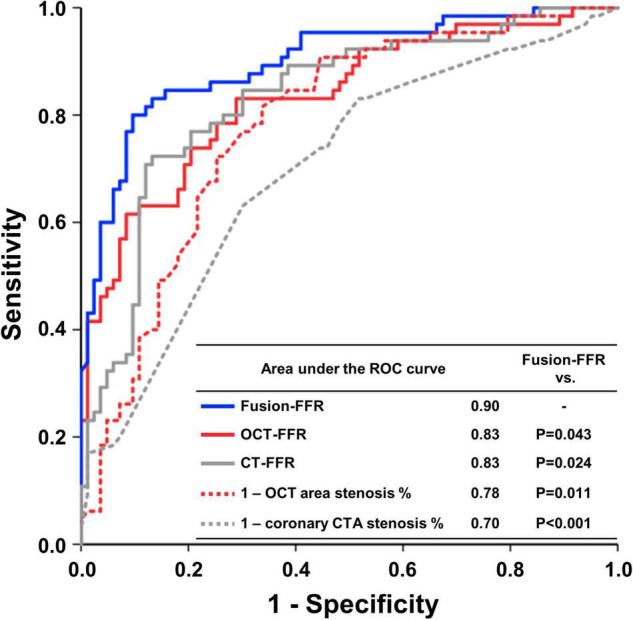
Receiver operating characteristics curves in assessing functionally significant stenosis for CFD-based computational FFRs and anatomic variables. Receiver operating characteristics (ROC) curves with area under the curve to assess functionally significant stenosis for Fusion-FFR, OCT-FFR, CT-FFR, percentage area stenosis on OCT, and percentage coronary CTA stenosis. CFD, computational fluid dynamics; CTA, computed tomography angiography; CT-FFR, computational FFR from coronary CTA; FFR, fractional flow reserve; Fusion-FFR, computational FFR from coronary CTA-OCT fusion images; OCT, optical coherence tomography; OCT-FFR, computational FFR from OCT.

The area under the ROC curve in assessing functionally significant stenosis is presented in Figure 4. The area was higher for Fusion-FFR than for CT-FFR (0.90 [95% CI: 0.84–0.94] vs. 0.83 [95% CI: 0.76–0.89], *P* = 0.024) and OCT-FFR (0.90 [95% CI: 0.84–0.94] vs. 0.83 [95% CI: 0.76–0.89], *P* = 0.043). The area was not different between CT-FFR and OCT-FFR (*P* = 0.947). The area was also higher for Fusion-FFR than for anatomic variables (percentage area stenosis on OCT: 0.78 [95% CI: 0.71–0.85]; percentage coronary CTA stenosis: 0.70 [95% CI: 0.62–0.77]). The diagnostic performance of computational FFRs in assessing functionally significant stenosis is presented in Table 3. Fusion-FFR exhibited 84.5% accuracy, 84.6% sensitivity, 84.3% specificity, 80.9% positive predictive value, and 87.5% negative predictive value. The diagnostic performance, especially accuracy, specificity, and positive predictive value were superior for Fusion-FFR, compared to those of CT-FFR (73.0%, *P* = 0.007; 61.4%, *P* < 0.001; 64.0%, *P* < 0.001) and OCT-FFR (75.7%, *P* = 0.021; 73.5%, *P* = 0.020; 69.9%, *P* = 0.012). The diagnostic performance was not different between CT-FFR and OCT-FFR, except for specificity which was superior for OCT-FFR (*P* = 0.041).

**TABLE 3:** Diagnostic performance of CFD-based computational FFRs in assessing functionally significant stenosis.

	Fusion-FFR	CT-FFR	OCT-FFR	*p*-value	
				Fusion-FFR vs. CT-FFR	Fusion-FFR vs. OCT-FFR
Accuracy	84.5 (77.0–89.9)	73.0 (65.2–78.3)	75.7 (67.5–82.3)	0.007	0.021
Sensitivity	84.6 (75.8–93.4)	87.7 (78.9–93.8)	78.5 (68.5–88.5)	0.527	0.248
Specificity	84.3 (76.5–92.2)	61.4 (54.6–66.2)	73.5 (64.0–83.0)	<0.001	0.020
Positive predictive value	80.9 (71.5–90.2)	64.0 (57.6–68.5)	69.9 (59.3–80.4)	<0.001	0.012
Negative predictive value	87.5 (80.3–94.7)	86.4 (76.7–93.2)	81.3 (72.5–90.2)	0.799	0.120

*Values are presented as % (95% confidence interval). CFD, computational fluid dynamics; CTA, computed tomography angiography; CT-FFR, computational FFR from coronary CTA; FFR, fractional flow reserve; Fusion-FFR, computational FFR from coronary CTA-OCT fusion images; OCT, optical coherence tomography; OCT-FFR, computational FFR from OCT.*

## Discussion

### Main Findings

This study presented a novel approach for estimating CFD-based computational FFR in patients with intermediate stenosis in the LAD, which was derived from coronary CTA-OCT fusion images. The main findings were as follows: (1) Fusion-FFR was strongly correlated with pressure wire-based FFR; although CT-FFR and OCT-FFR were also well correlated with FFR, Fusion-FFR was more strongly correlated; (2) area under the ROC curve in assessing functionally significant stenosis was higher for Fusion-FFR than for CT-FFR and OCT-FFR; and (3) the diagnostic performance, especially accuracy, specificity, and positive predictive value of Fusion-FFR were superior to those of CT-FFR and OCT-FFR.

### Clinical Implications of Computational Fractional Flow Reserve From Fusion Images

Deciding whether to proceed with coronary revascularization is difficult, and simple angiographic assessment of luminal narrowing has led to a misdiagnosis rate as high as 40% (1, 2, 25). Therefore, functional assessment of coronary stenosis by FFR measurement is important to make appropriate decisions regarding coronary revascularization, especially in patients with intermediate stenosis (1–7). Although coronary CTA and OCT were originally developed for anatomical evaluation of coronary vessels and plaques, recent techniques have applied CFD to estimate computational FFR from three-dimensional coronary models derived from these images and demonstrated favorable results (11–14). In addition, current advances in image reconstruction techniques have also enabled more precise three-dimensional model reconstruction *via* the fusion of images from different imaging tools (26). Coronary CTA allows detailed visualization of vessel curvature but has the disadvantage of low resolution; in contrast, OCT produces exceptionally high-resolution images but does not allow sufficient visualization of vessel curvature (8–10). Therefore, fusing images from both methods is expected to provide more reliable information about the coronary stenotic lesions by combining detailed vessel curvature data from coronary CTA with accurate lumen contour data from OCT. Since the excellent resolution of OCT is widely known, we estimated several CFD-based flow characteristics to support our hypothesis regarding the contribution of vessel curvature of coronary CTA toward fusion images (10, 13). Coronary CTA and fusion images showed higher vorticity, helicity, and wall shear stress than those of OCT with insufficient visualization of vessel curvatures (Supplementary Figure 3). To the best of our knowledge, this is the first study to investigate CFD-based computational FFR using coronary CTA-OCT fusion images in patients with intermediate stenosis, and our technique has shown promising results. Fusion-FFR was strongly correlated with pressure wire-based FFR, had a high area under the ROC curve in assessing functionally significant stenosis, and exhibited good diagnostic performance.

### Fusion-Fractional Flow Reserve vs. Coherence Tomography-Fractional Flow Reserve and Optical Coherence Tomography-Fractional Flow Reserve

According to the DISCOVER-FLOW (Diagnosis of Ischemia-Causing Stenoses Obtained *Via* Non-invasive Fractional Flow Reserve) study, CT-FFR and pressure wire-based FFR were well correlated (*r* = 0.717, *P* < 0.001), and the area under the ROC curve for detecting ischemia was higher for CT-FFR than for coronary CTA stenosis (0.90 vs. 0.75, *P* = 0.001) (11). Superior diagnostic performance of CT-FFR vs. coronary CTA stenosis was also reported in the NXT (Analysis of Coronary Blood Flow Using CT Angiography: Next Steps) trial with a higher area under the ROC curve for detecting ischemia (0.90 vs. 0.81, *P* < 0.001) (12). However, use of CT-FFR is limited by the low resolution of coronary CTA images (13, 27). In contrast, OCT provides high-resolution images, thereby supplying more precise information regarding coronary arteries and atherosclerotic plaques (10). In their first study estimating OCT-FFR using CFD, Ha et al. found that OCT-FFR and pressure wire-based FFR were well correlated (*r* = 0.72, *P* < 0.001), and the area under the ROC curve for detecting ischemia was high (0.93) (13). Similarly, Yu et al. also reported that OCT-FFR and pressure wire-based FFR were well correlated (*r* = 0.70, *P* < 0.001), and the area under the ROC curve for detecting ischemia was higher for OCT-FFR than for area stenosis on OCT (0.93 vs. 0.80, *P* = 0.002) (14).

Although previous approaches for estimating computational FFR from coronary CTA or OCT were innovative, they used only a single tool. Currently, OCT is widely used to visualize the microstructures of coronary stenotic lesions and plaques, and many patients undergo coronary CTA to assess suspected coronary artery disease before being referred for angiography (9, 10, 15). Thus, we hypothesized that if we encounter intermediate stenosis during angiography, which needs further anatomical or functional assessment, OCT can provide not only precise anatomical information based on high resolutions but also functional information based on CFD (OCT-FFR) without using additional guide wires or hyperemic agents. In addition, if the patients have already undergone coronary CTA for evaluation of chest pain before angiography, we might be able to fuse the coronary CTA and OCT images to obtain more reliable, functional information regarding coronary stenosis (Fusion-FFR). In this study, although computational FFR estimated from the single modality of coronary CTA or OCT was well correlated with FFR, Fusion-FFR showed a stronger correlation with FFR and exhibited a significantly higher area under the ROC curve in assessing functionally significant stenosis. Likewise, the diagnostic performance, especially accuracy, specificity, and positive predictive value of Fusion-FFR, were superior to those of CT-FFR and OCT-FFR. Our findings regarding the benefits of fusing images from different imaging tools are reflective of improved lumen size determination and hemodynamic assessment using a three-dimensional fusion model of three-dimensional QCA and OCT, compared with three-dimensional QCA alone (26). Thus, our findings suggest that computational FFR from fusion images of two different imaging tools, coronary CTA and OCT, may be more valuable than computational FFR based on a single tool in the setting of intermediate coronary stenosis, especially for excluding functionally non-significant stenosis and consequently for reducing unnecessary revascularization procedures.

### Further Applications of Coronary Optical Coherence Tomography—Optical Coherence Tomography Fusion Images

To overcome the limitations of CFD, previous studies have demonstrated the possibility of on-site application of CT-FFR based on machine learning with the advantage of high processing speed. However, its performance was limited by the image quality or calcium burden (28, 29). In this regard, in patients with both coronary CTA and OCT images, machine learning may be applied to coronary CTA-OCT fusion images, which contain data not only from coronary CTA but also from OCT with high-resolution images, to overcome the limitations of machine learning-based CT-FFR and enhance the on-site application of Fusion-FFR technique in real-world clinical practice. In addition, recent studies have shown that machine learning algorithms can accurately detect anatomical features on OCT, such as thin-cap fibroatheroma, as well as identify relevant anatomical features on coronary CTA, which are associated with present and future ischemic events (30, 31). Efficiency and cost savings are improved in clinical practice by making full use of available diagnostic modalities. Thus, future studies based on machine learning to explore the effect of anatomical and functional information from coronary CTA-OCT fusion images to identify current myocardial ischemia and predict future cardiovascular events are expected.

### Study Limitations

This study has some limitations. First, as a retrospective analysis of a prospectively enrolled registry, it has the inherent limitations of the current study design. Furthermore, although statistical significance was found for superior diagnostic performance of Fusion-FFR vs. CT-FFR and OCT-FFR, the study population was relatively small. Second, we assessed FFR alone in LAD lesions to reduce possible confounding factors. Besides, we chose this artery due to its clinical importance, and the correlation between anatomical and functional indices is better for the LAD than for other coronary arteries (13, 32). Third, we removed the side branches while reconstructing the three-dimensional coronary model to simplify the process for CFD. Although this study focused on combining detailed vessel curvature from coronary CTA with accurate lumen contour from OCT and demonstrated promising results, the effects of side branches on computational FFR require further investigation. Fourth, in total, 14 patients (7.8%) were excluded due to poor quality of coronary images or incomplete OCT coverage. Fifth, the cut-off value ≤ 0.80 was used not only for pressure wire-based FFR but also computational FFRs in non-hyperemic condition to define functionally significant stenosis. Sixth, microvascular resistance was not assessed in this study, therefore, the effect of microvascular dysfunction on functional assessment of coronary stenosis could not be evaluated. Nevertheless, the current study results are innovative and warrant further larger population-based prospective studies, including all coronary vessels and side branches, to assess the real-world clinical applicability of the Fusion-FFR technique in patients with both coronary CTA and OCT images. In addition, machine learning may be applied to coronary CTA-OCT fusion images to enhance the on-site application of Fusion-FFR technique during the angiographic procedure.

## Conclusion

A novel approach of estimating computational FFR from coronary CTA-OCT fusion images provided more accurate functional information than FFR computed from coronary CTA or OCT alone.

## Data Availability Statement

The original contributions presented in this study are included in the article/Supplementary Material, further inquiries can be directed to the corresponding authors.

## Ethics Statement

The studies involving human participants were reviewed and approved by the Institutional Review Board of Severance Cardiovascular Hospital, Keimyung University Dongsan Hospital, Yeungnam University Medical Center, and National Health Insurance Service Ilsan Hospital. The patients/participants provided their written informed consent to participate in this study.

## Author Contributions

Y-JL, YK, and JH did the data analyses and wrote the original draft. All authors interpreted the results, contributed to the critical revising of the manuscript, and approved the final version of the manuscript for submission.

## Conflict of Interest

GG was a consultant in St. Jude Medical and has received institutional research grants. The remaining authors declare that the research was conducted in the absence of any commercial or financial relationships that could be construed as a potential conflict of interest.

## Publisher’s Note

All claims expressed in this article are solely those of the authors and do not necessarily represent those of their affiliated organizations, or those of the publisher, the editors and the reviewers. Any product that may be evaluated in this article, or claim that may be made by its manufacturer, is not guaranteed or endorsed by the publisher.
