# Supplementary material for: Computational Fractional Flow Reserve From Coronary Computed Tomography Angiography—Optical Coherence Tomography Fusion Images in Assessing Functionally Significant Coronary Stenosis
Source: Front Cardiovasc Med. 2022 Jun 13;9:925414. doi: 10.3389/fcvm.2022.925414 (PMC9234158; doi:10.3389/fcvm.2022.925414)
Supplement: Supplementary file 1 [file Data_Sheet_1.pdf]

Supplementary Figure 1. Flowchart of study subjects

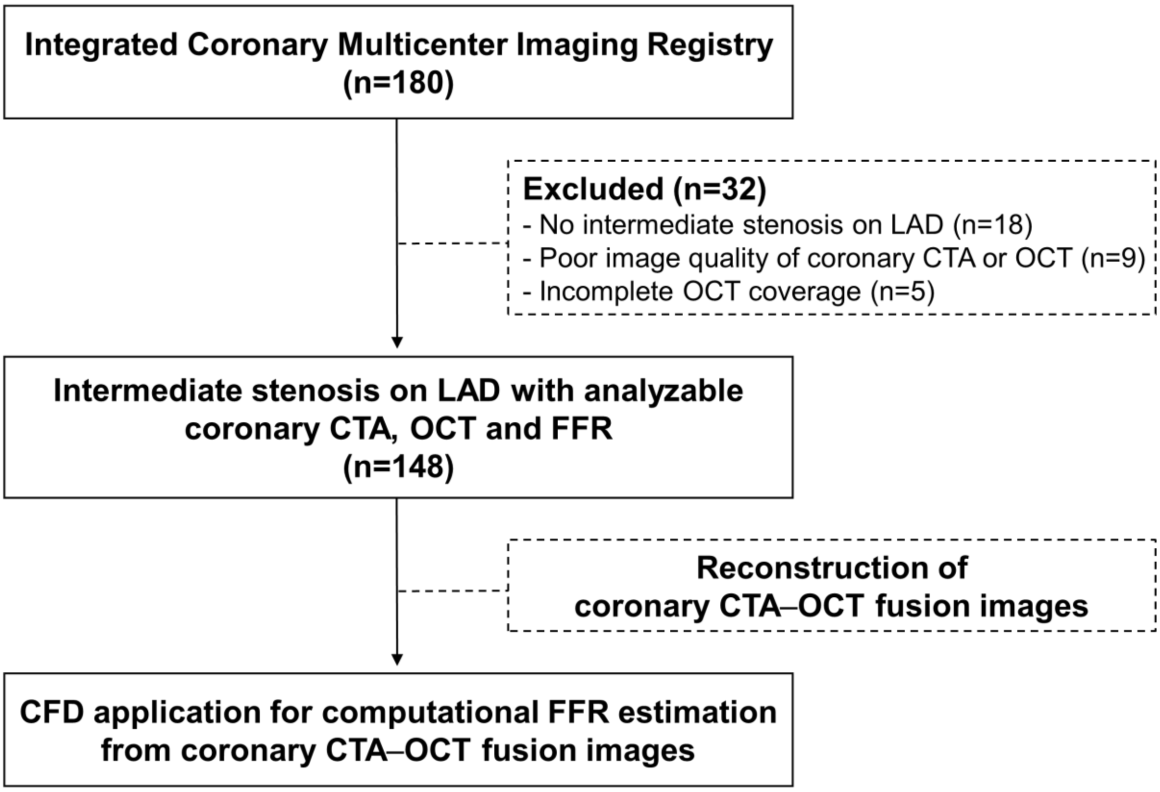

CFD, computational fluid dynamics; CTA, computed tomography angiography; FFR, fractional flow reserve; LAD, left anterior descending artery; OCT, optical coherence tomography.

Supplementary Figure 2. Agreement between pressure wire-based FFR and CFD-based computational FFR from coronary CTA or OCT images

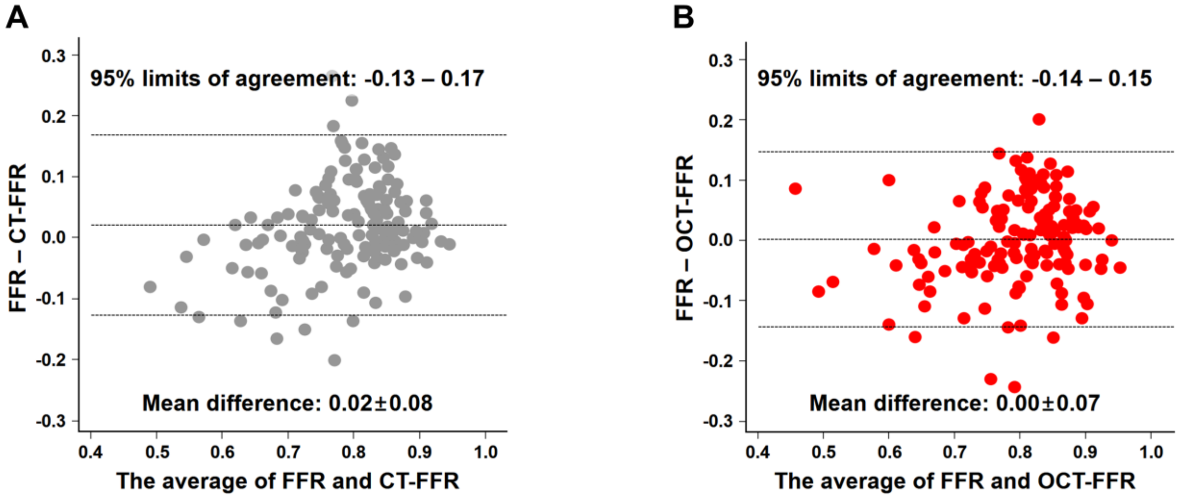

Agreement between pressure wire-based FFR and CT-FFR **(A)**, and between FFR and OCT-FFR **(B)**.

CFD, computational fluid dynamics; CTA, computed tomography angiography; CT-FFR, computational FFR from coronary CTA; FFR, fractional flow reserve; OCT, optical coherence tomography; OCT-FFR, computational FFR from OCT.

Supplementary Figure 3. Comparison of CFD-based flow characteristics between coronary CTA, OCT, and coronary CTA-OCT fusion images

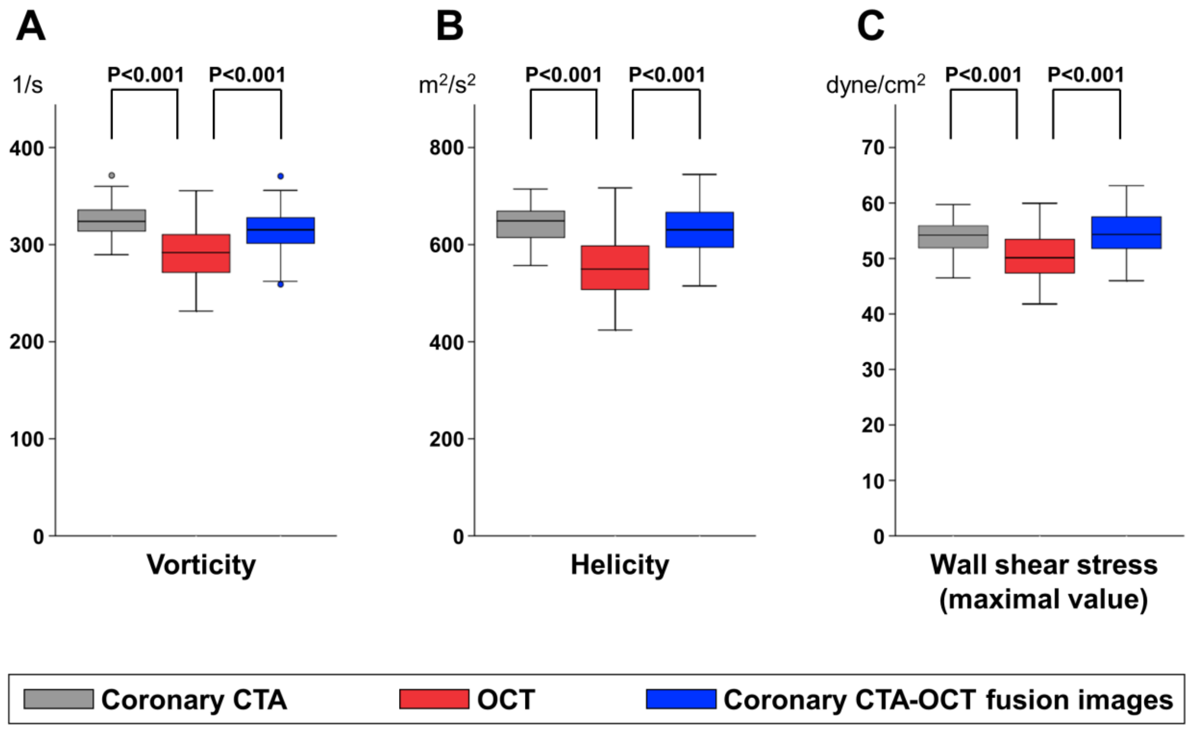

Vorticity **(A)**, helicity **(B)**, and wall shear stress **(C)** were estimated and compared to support our hypothesis regarding the contribution of vessel curvature of coronary CTA toward fusion images.

CFD, computational fluid dynamics; CTA, computed tomography angiography; OCT, optical coherence tomography.

Supplementary Table 1. Inclusion and exclusion criteria for Integrated Coronary Multicenter Imaging Registry

Inclusion criteria

1. Patients who underwent coronary CTA for chest pain
2. Age 20-80 years
3. Intermediate diameter stenosis (40%–70%) on coronary angiography at de novo lesions in the proximal to middle portion of any coronary artery which requires pressure wire-based FFR measurement or OCT examination

Exclusion criteria

1. Requirement for inotropics for hemodynamic instability
2. Severe left ventricular dysfunction (ejection fraction <30%)
3. Severe valvular dysfunction
4. Declined kidney dysfunction (serum creatinine >2.0 mg/dL)
5. Contraindication to adenosine or contrast
6. Expected survival less than 12 months
7. Inability to follow the patient over the period of 2 years after enrollment, as assessed by the investigator
8. Inability to understand or read the informed consent

CTA, computed tomography angiography; FFR, fractional flow reserve; OCT, optical coherence tomography.

Supplementary Table 2. Definition of plaque characteristics on optical coherence tomography (1-3)

Characteristics	Definition
**Fibrous plaque**	Plaque with high backscattering and a relatively homogeneous signal
**Fibrocalcific plaque**	Plaque which contains fibrous tissue with calcium that appears as a heterogeneous or signal-poor region with sharply delineated border
**Lipid plaque**	Plaque with diffusely bordered signal-poor region overlain by signal rich band
**Intimal vasculature**	Small, signal-poor regions with vesicular or tubular shape within the intima without a connection to the vessel lumen which can usually be followed in continuous frames
**Cholesterol crystal**	Thin, linear and back scattering regions of high intensity
**Calcific nodule**	Single or multiple regions of calcium which protrude into the lumen, frequently forming sharp and jutting angles

Supplementary References

1. Tearney GJ, Regar E, Akasaka T, Adriaenssens T, Barlis P, Bezerra HG, et al. Consensus standards for acquisition, measurement, and reporting of intravascular optical coherence tomography studies: a report from the International Working Group for Intravascular Optical Coherence Tomography Standardization and Validation. *J Am Coll Cardiol.* (2012) 59:1058-72. doi: 10.1016/j.jacc.2011.09.079.
2. Prati F, Regar E, Mintz GS, Arbustini E, Di Mario C, Jang IK, et al. Expert review document on methodology, terminology, and clinical applications of optical coherence tomography: physical principles, methodology of image acquisition, and clinical application for assessment of coronary arteries and atherosclerosis. *Eur Heart J.* (2010) 31:401-415. doi: 10.1093/eurheartj/ehp433.
3. Vergallo R, Porto I, D'Amario D, Annibali G, Galli M, Benenati S, et al. Coronary Atherosclerotic Phenotype and Plaque Healing in Patients With Recurrent Acute Coronary Syndromes Compared With Patients With Long-term Clinical Stability: An In Vivo Optical Coherence Tomography Study. *JAMA Cardiol.* (2019) 4:321-329. doi: 10.1001/jamacardio.2019.0275.
